# Cost-effectiveness of diagnostic laparoscopy for assessing resectability in pancreatic and periampullary cancer

**DOI:** 10.1186/s12876-015-0270-x

**Published:** 2015-04-02

**Authors:** Stephen Morris, Kurinchi S Gurusamy, Jessica Sheringham, Brian R Davidson

**Affiliations:** 1 Department of Applied Health Research, University College London, Gower Street, 1-19 Torrington Place, London, WC1E 7HB UK; 2 University College London Medical School, 9th Floor, Royal Free Hospital, Rowland Hill Street, London, UK

**Keywords:** Cost-effectiveness analysis, Diagnostic laparoscopy, Pancreatic cancer, Periampullary cancer

## Abstract

**Background:**

Surgical resection is the only curative treatment for pancreatic and periampullary cancer, but many patients undergo unnecessary laparotomy because tumours can be understaged by computerised tomography (CT). A recent Cochrane review found diagnostic laparoscopy can decrease unnecessary laparotomy. We compared the cost-effectiveness of diagnostic laparoscopy prior to laparotomy versus direct laparotomy in patients with pancreatic and periampullary cancer with resectable disease based on CT scanning.

**Method:**

Model based cost-utility analysis estimating mean costs and quality-adjusted life years (QALYs) per patient from the perspective of the UK National Health Service. A decision tree model was constructed using probabilities, outcomes and cost data from published sources. One-way and probabilistic sensitivity analyses were undertaken.

**Results:**

When laparotomy following diagnostic laparoscopy occurred in a subsequent admission, diagnostic laparoscopy incurred similar mean costs per patient to direct laparotomy (£7470 versus £7480); diagnostic laparoscopy costs (£995) were offset by avoiding unnecessary laparotomy costs. Diagnostic laparoscopy produced significantly more mean QALYs per patient than direct laparotomy (0.346 versus 0.337). Results were sensitive to the accuracy of diagnostic laparoscopy and the probability that disease was unresectable. Diagnostic laparoscopy had 63 to 66% probability of being cost-effective at a maximum willingness to pay for a QALY of £20 000 to £30 000. When laparotomy was undertaken in the same admission as diagnostic laparoscopy the mean cost per patient of diagnostic laparoscopy increased to £8224.

**Conclusions:**

Diagnostic laparoscopy prior to laparotomy in patients with CT-resectable cancer appears to be cost-effective in pancreatic cancer (but not in periampullary cancer), when laparotomy following diagnostic laparoscopy occurs in a subsequent admission.

**Electronic supplementary material:**

The online version of this article (doi:10.1186/s12876-015-0270-x) contains supplementary material, which is available to authorized users.

## Background

Surgical resection is generally considered to be the only curative treatment for pancreatic and periampullary cancer (which includes ampullary cancer and duodenal cancer along with cancer of the head of the pancreas). Only 15 to 20% of patients undergo potentially curative resection [1-5]. In the remaining patients, the tumours are not resectable because the cancer has spread into surrounding structures or because of disseminated disease. Despite the availability of high quality imaging including helical computed tomography (CT scanning), endoscopic ultrasound (EUS), and magnetic resonance imaging (MRI), 25% to 40% of patients who undergo laparotomy for pancreatic head cancer/periampullary cancer could not be resected, with non-resectability identified only during laparotomy [6,7].

A recent Cochrane Review of 15 studies and a total of 1015 patients found that diagnostic laparoscopy prior to laparotomy can decrease the rate of unnecessary laparotomy from 40% to 17% in patients with pancreatic and periampullary cancer found to have resectable disease from a CT scan [8]. Diagnostic or staging laparoscopy is still relevant therefore to detect metastases not identified by high quality imaging techniques such as CT scanning. The Cochrane Review included only studies in which biopsy confirmation of metastatic spread was obtained. The specificity of diagnostic laparoscopy in all studies was 1, since the review included only studies in which diagnostic laparoscopy along with biopsy confirmation of metastatic spread was used as the index test. A review of the NHS Economic Evaluations Database [9] using the search term (laparoscop*) AND ((pancrea*) OR (periampull*)) [28 August 2013] identified 31 studies, but none of these evaluated diagnostic laparoscopy in patients who were resectable following CT scanning. This study investigates the cost-effectiveness of diagnostic laparoscopy prior to laparotomy versus direct laparotomy in patients with pancreatic and periampullary cancer who were considered to have resectable disease and be suitable for major surgery following CT scanning.

## Methods

This is a model-based cost-utility analysis to estimate the mean cost per patient and the mean outcome per patient associated with diagnostic laparoscopy prior to laparotomy versus direct laparotomy in patients with pancreatic or periampullary cancers, found to have resectable disease from a CT scan. In our base case we assume that laparotomy following diagnostic laparoscopy occurs in a subsequent admission, and therefore if the laparotomy is unnecessary there is a cost saving because use of the operating theatre and the hospital stay are avoided. In a sensitivity analysis we consider a situation where the laparotomy is undertaken in the same admission as the diagnostic laparoscopy. In this case we assume the cost saving is smaller because while the hospital stay is avoided the cost of the operating theatre would still be incurred if the laparotomy is cancelled.

The outcome measure is quality-adjusted life years (QALYs), which combine length of life and quality of life [10]. QALYs are the recommended outcomes for use in economic evaluations in the UK as they are a common unit that allow for comparable decisions about resource allocation across different health conditions. The analysis is undertaken from the perspective of the UK National Health Service (NHS). Costs are calculated in 2011/12 UK£. Since diagnostic laparoscopy is unlikely to affect long term disease outcomes, a time horizon of six months for costs and outcomes was considered to be appropriate. This is sufficiently long to capture the negative impact of laparotomy on quality of life [11-13]. Due to the short time horizon, discounting of costs and benefits was unnecessary.

### Model structure

**Figure 1:**
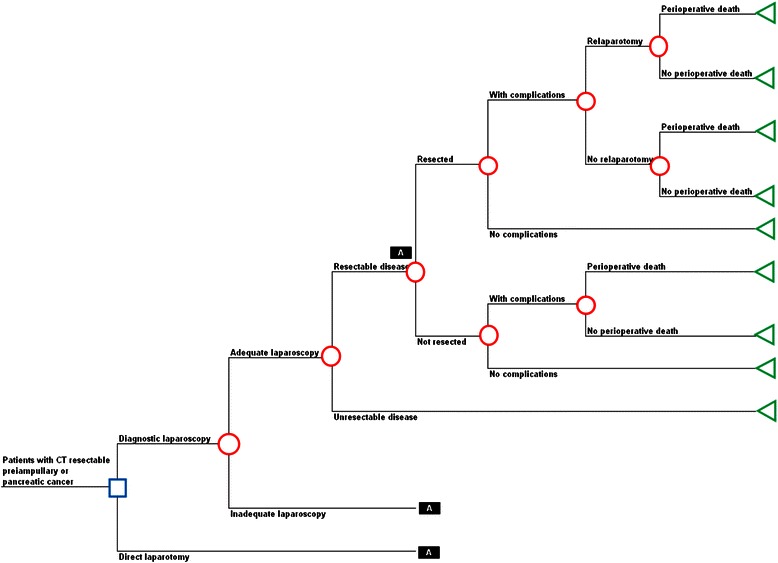
**Decision tree model structure.**

The analysis uses a decision tree to describe the options being compared and the possible pathways following them (Figure 1). This is a commonly used approach in cost-effectiveness studies of health care programmes [10]. The nodes of a decision tree are points where more than one event is possible. The branches are mutually exclusive events following each node. Decision nodes, represented by squares, show the different options that might be chosen by decision-makers based on the costs and benefits they produce (e.g., to choose diagnostic laparoscopy or direct laparotomy). Chance nodes, represented by circles, show uncertain events, each of which is associated with a probability that it will occur (e.g., whether the diagnostic laparoscopy will show that the cancer is resectable or not). Terminal nodes, represented by triangles, are the endpoints of a decision tree, beyond which no further pathways are available. Each terminal node has costs and QALYs associated with it, summarising the sequence of decisions and events on a unique path leading from the initial decision node to that terminal node. These costs and QALYs are expected values, based on the probability of each event on the pathway occurring up to that point and the costs and QALYs associated with each event.

Patients enter the model with pancreatic or periampullary cancer that has been identified as being resectable following CT scanning. If they undergo diagnostic laparoscopy this may be adequate for determining resectability if histologic confirmation of metastatic disease is possible. If the diagnostic laparoscopy is adequate then it will indicate whether or not the tumour is resectable and if it is, the patient will have a laparotomy. During the laparotomy the tumour may be resected or not. If it is not resected, the patient receives palliative treatment. The laparotomy may result in complications, in some cases an additional laparotomy may be required to treat the complications, and the patient may die perioperatively. If the laparoscopy identifies the tumour as not being resectable then curative surgery is not undertaken and the patient will receive palliative treatment.

For patients undergoing direct laparotomy, it was assumed that the pathway was the same as for resectable disease being identified after adequate laparoscopy, but the probabilities, costs and QALYs associated with each pathway may be different. If the diagnostic laparoscopy was inadequate for histologic confirmation of metastatic disease, the diagnostic laparoscopy was considered to be non-informative and the subsequent pathway was assumed to be as for direct laparotomy but also incurring the costs of the diagnostic laparoscopy procedure.

### Probabilities

The probabilities associated with mutually exclusive events at each chance node were obtained from published sources (Additional file 1) [8,14,15]. The probability of non-resectable disease with direct laparotomy was 0.403, calculated in the Cochrane Review as the median pre-test prevalence after CT scan of unresectable disease due to distant metastases or local infiltration [8]. Values in the individual studies included in the Cochrane Review ranged from 0.17 to 0.82 [8]. The Cochrane Review also calculated a post-test probability of unresectable disease of 0.173 (95% CI 0.12 to 0.24), meaning that if a patient is said to have resectable disease after diagnostic laparoscopy, there is a 0.173 probability that their cancer will be unresectable. The difference in the probability that the tumour is unresectable following adequate diagnostic laparoscopy compared with direct laparotomy is therefore 0.403-0.173 = 0.230, meaning that on average using diagnostic laparoscopy prior to laparotomy would avoid 230 unnecessary laparotomies in 1000 patients in whom laparotomy is planned for curative resection of pancreatic cancers [8] and 770 patient would have a laparotomy. The probability of undergoing laparotomy is 1–0.230 = 0.770, and the probability the tumour is unresectable among those who have a laparotomy is 0.173/0.770 = 0.225. Put another way, with direct laparotomy, 403 patients in 1000 would have unresectable disease. With diagnostic laparoscopy 230 of these patients would avoid an unnecessary laparotomy, 770 would have a laparotomy and 770*0.225 = 173 of these would have unresectable disease. The probabilities of complications with laparotomy, of relaparotomy, and of perioperative death were taken from a cohort study of 366 patients with pancreatic cancer [14]. The probability of inadequate laparoscopy was taken from one of the studies included in the Cochrane Review which reported this information [15].

### Outcomes

QALYs combine length of life and quality of life, where the latter is measured by utility scores. A utility score of 1 represents full health and a utility of 0 death; negative values represent states worse than death. A review of the NHS Economic Evaluations Database [9] was undertaken using the search terms (pancrea* OR ampullary OR periampullary) AND (QALY) [23 February 2014] to identify studies reporting relevant utility scores. After reviewing the reference lists of the identified studies and removing duplicates, 5 studies containing potentially relevant utility data were identified [16-20]. The utility scores used in the model were from one study [19], selected because values were presented for different points over time, because utility scores for all the health states in the model were included in this study enabling better comparability between values, and the values reported also reflected trends in disease-specific quality of life measures found in other studies [11-13] (Additional file 1). Utility scores were measured at 2 weeks, 3 months and 6 months. QALYs were estimated using the trapezium rule for calculating the area under the curve.

### Costs

The cost of diagnostic laparoscopy, including histological examination of tissue obtained at laparoscopy was assumed to be £995 (Additional file 1) [21]. This is the average value of the elective inpatient and day case cost, weighted by the proportion of patients in each group. Surgical resection with and without complications was assumed to cost £12 006 and £7083, respectively [21]. Laparotomy without resection was assumed to cost £5378 with complications and £4487 without complications [21]. The cost of repeat laparotomy was assumed to be £7083 [21].

### Measuring cost-effectiveness

Cost-effectiveness was measured using monetary net benefits (MNBs). For each treatment the MNB was calculated as the mean QALYs per patient accruing to that treatment multiplied by decision-makers’ maximum willingness to pay for a QALY (also referred to as the cost-effectiveness threshold, which in the UK is approximately £20 000 to 30 000 per QALY gained [22]), minus the mean cost per patient for the treatment. This approach converts the outcomes from each treatment into monetary terms and then subtracts the costs of each treatment from the monetised benefits, calculating the net benefit of each treatment in monetary terms. MNBs were calculated using the base case parameter values shown in Additional file 1; these are referred to as the deterministic results since they do not depend on chance. The treatment with the highest MNB represents good value for money and is preferred on cost-effectiveness grounds.

### Sensitivity analyses

One-way sensitivity analysis was undertaken, varying the probabilities, outcomes and costs one at a time within the ranges listed in Additional file 1. The aim was to identify the threshold value for each parameter, where one exists, where the treatment with the highest MNB changed (e.g., the value at which diagnostic laparoscopy was no longer the most cost-effective option).

We undertook a probabilistic sensitivity analysis (PSA) as recommended by the National Institute for Health and Care Excellence (NICE) [22]. Distributions were assigned to parameters (Additional file 1) to reflect the uncertainty with each parameter value. A random value from the corresponding distribution for each parameter was selected. This generated an estimate of the mean cost and mean QALYs and the MNB associated with each treatment. This was repeated 5000 times and the results for each simulation were noted. The mean costs, QALYs and MNBs for each treatment were calculated from the 5000 simulations; these are referred to as the probabilistic results since they depend on chance. Using the MNBs for each of the 5000 simulations the proportion of times each treatment had the highest MNB was calculated for a range of values for the maximum willingness to pay for a QALY. These were summarised graphically using cost-effectiveness acceptability curves [10].

In the PSA we used beta distributions to model uncertainty in the probabilities and utility scores, and gamma distributions to model uncertainty in costs [23]. In cases where standard errors were required for the PSA and these were not reported in the sources used it was assumed the standard error was equal to the mean [23]. For the probability of unresectable disease with direct laparotomy after CT scanning, the parameter values for the beta distribution were based on the numbers of unresectable and resectable cancers pooled across all studies included in the Cochrane Review. For the post-test probability of unresectable disease the parameter values were calculated from the 95% confidence interval reported in the Cochrane Review. For the utilities the variance was calculated assuming a beta distribution based on 97 observations [19,20]. 95% confidence intervals around the base case values were derived using standard deviations calculated from the 5000 simulations in the PSA.

We undertook a further sensitivity analysis to investigate the cost savings associated with diagnostic laparoscopy. We considered a situation where the laparotomy following diagnostic laparoscopy was scheduled for the same admission as the diagnostic laparoscopy. When the diagnostic laparoscopy indicated the tumour was not resectable, so the laparotomy was not required, the cost of the hospital stay was avoided but the cost of the operating theatre time was not. This was assumed to cost £3524, based on 4 hours of theatre time at £881 per hour [24].

Finally, because of jaundice being a relative early presentation of ampullary cancers, the resectability rate of ampullary cancers are believed to be higher than that of pancreatic cancers [25]. We therefore reran our analyses separately based on studies from the Cochrane Review that included only patients with pancreatic cancer and only patients with periampullary cancer. As shown in the Cochrane Review, for patients with pancreatic cancer the sensitivity of diagnostic laparoscopy was 67.9%, the median pre-test probability of unresectability was 0.400 and the post-test probability of unresectable disease after negative diagnostic laparoscopy was 0.180. One study in the Cochrane Review included only patients with periampullary cancer [15]. In this study of 144 patients the sensitivity of diagnostic laparoscopy was 52.0%, the pre-test probability of unresectability was 0.174 and the post-test probability of unresectable disease after negative diagnostic laparoscopy was 0.092. We reran our models using these two sets of values holding all other values constant.

## Results

Using base case values, and assuming laparotomy following diagnostic laparoscopy occurs in a subsequent admission, diagnostic laparoscopy prior to resection incurred similar costs as proceeding straight to laparotomy without prior laparoscopy (mean cost per patient £7470 (95% CI £7215 to £7724) versus £7480 (95% CI £7219 to £7741) (Table 1); the cost of the diagnostic laparoscopy (£995) was offset by avoiding the costs of unnecessary laparotomy. QALYs up to 6 months were higher for diagnostic laparoscopy compared with direct laparotomy (mean QALYs per patient 0.346 (95% CI 0.346 to 0.347) versus 0.337 (95% CI 0.337 to 0.338)) due to the negative impact of unnecessary laparotomy.

**Table 1:** **Base case results**

	Diagnostic laparoscopy	Direct laparotomy
Costs	7470 (7356, 7583)	7480 (7363, 7597)
QALYs	0.346 (0.346, 0.347)	0.337 (0.337, 0.338)
Monetary net benefit		
£20 000	−543 (−429, −656)	−738 (−621, −855)
£30 000	2921 (2807, 3035)	2633 (2516, 2750)

QALY = quality adjusted life year. Costs are in 2011/12 UK£. Figures are expected values per patient with 95% confidence intervals in brackets. The point estimates are calculated using base case values of the model parameters (deterministic results). The 95% confidence intervals are derived using standard deviations calculated from the 5000 simulations in the probabilistic sensitivity analysis. The monetary net benefit is calculated at a maximum willingness to pay for a QALY of £20 000 and £30 000. The results are calculated using base case values of the model parameters. Numbers may not sum due to rounding.

The MNB for diagnostic laparoscopy prior to laparotomy was significantly higher than those for direct laparotomy at a maximum willingness to pay for a QALY of £30 000 (£2921 (95% CI £2807 to £3035) versus £2633 (95% CI £2516 to £2750)) but at a willingness to pay for a QALY of £20 000 the MNB for diagnostic laparoscopy was numerically higher but the 95% CIs overlapped. As expected, the probabilistic results (not shown) were numerically similar to the deterministic results.

In the one-way sensitivity analysis the results were sensitive to changing the values of probability of non-resectable disease with direct laparotomy: values in the individual studies included in the Cochrane Review ranged from 0.17 to 0.82 [8]; for values <0.36 direct laparatomy had the highest MNB. Results were also sensitive to the post-test probability of unresectable disease: the Cochrane review calculated that the 95% CI of this probability was 0.12 to 0.24 [8]; at values > 0.22 direct laparatomy had the highest MNB.

**Figure 2:**
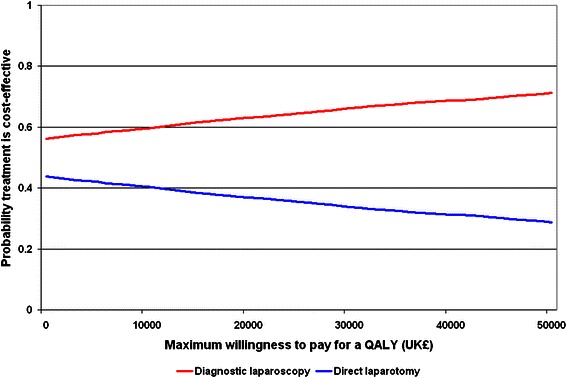
**Cost-effectiveness acceptability curves.** The acceptability curves show the probability that each option is cost-effective at different values of the maximum willingness to pay for a quality adjusted life year (QALY).

The cost-effectiveness acceptability curves for each treatment show that diagnostic laparoscopy prior to laparotomy had a 63.2% probability of being cost-effective at a maximum willingness to pay for a QALY of £20 000 and a 66.2% probability at a value of £30 000 (Figure 2).

When laparotomy was scheduled for the same admission as diagnostic laparoscopy, and the cost of the hospital stay was avoided if the tumour was unresectable but the cost of the operating theatre time was not, the costs avoided by unnecessary laparotomy were smaller and the mean cost per patient of diagnostic laparoscopy prior to resection increased from £7470 to £8224, which was higher than the cost of direct laparotomy. The MNB for diagnostic laparoscopy prior to laparotomy was lower than the MNB for direct laparotomy at a willingness to pay for a QALY of both £20 000 and £30 000 (−£1297 versus -£738 and £2167 versus £2633, respectively).

When we reran our analyses separately for subgroup of studies from the Cochrane Review that included only patients with pancreatic cancer the MNB for diagnostic laparoscopy prior to laparotomy was higher than the MNB for direct laparotomy at a willingness to pay for a QALY of both £20 000 and £30 000 (−£607 versus -£751 and £2853 versus £2621, respectively). When we reran our analyses separately for patients with periampullary cancer the MNB for diagnostic laparoscopy prior to laparotomy was lower that for direct laparotomy (−£2263 versus -£1693 and £1197 versus £1734, respectively).

## Discussion

### Main findings

We estimated the mean cost per patient and the mean outcome per patient associated with diagnostic laparoscopy prior to resection versus direct laparotomy in patients with pancreatic and periampullary cancer found to be resectable with curative intent following CT scanning. Diagnostic laparoscopy incurred the same overall costs as direct laparotomy (mean cost per patient £7470 versus £7480) and QALYs up to 6 months were slightly higher for diagnostic laparoscopy (mean QALYs per patient 0.346 versus 0.337) due to the avoidance of unnecessary laparotomy in patients with unresectable disease. The MNBs for diagnostic laparoscopy prior to resection were significantly higher than those for direct laparotomy at a maximum willingness to pay for a QALY of £30 000 but not £20 000. There is some uncertainty with this finding, with the results being sensitive to key model parameters for test accuracy (probability of unresectable disease (with curative intent) following CT scan that shows resectable disease, post-test probability of unresectable disease).

Diagnostic laparoscopy can either be performed as a separate procedure or immediately prior to laparotomy as part of larger procedure. The advantage of performing diagnostic laparoscopy as part of a larger procedure are that the patient needs only one hospital admission and one general anaesthetic. However if the patient is diagnosed as having unresectable disease at laparoscopy and the subsequent laparotomy is then cancelled, it means that operating time is wasted. If laparoscopy is performed as a separate diagnostic procedure, the patient must undergo the burden of two separate hospital admissions, time to surgery is delayed, which may increase the probability of unresectable disease, and anaesthetics but no operating time will be wasted if they are found to have unresectable disease. When laparotomy is undertaken in the same admission as diagnostic laparoscopy and the cost of the hospital stay is avoided but the cost of the operating theatre time is not, the costs of diagnostic laparoscopy are higher than those for direct laparotomy and the MNBs are lower. Diagnostic laparoscopy is not cost-effective in this scenario.

Diagnostic laparoscopy prior to laparotomy was cost-effective among patients with pancreatic cancer. It was not cost-effective in patients with periampullary cancer despite decreasing the unresectability from 17.4% to 9.2%. This subgroup analysis is based on a single study. One possible explanation of the finding is that fewer periampullary cancer patients had unresectable disease after a CT scan – 17.4% patients [15] compared to 40.0% patients [8]. As indicated in the one-way sensitivity analysis, the results of cost-effectiveness were sensitive to the proportion of patients with unresectable disease after a direct laparotomy. Since fewer unnecessary laparotomies were performed in patients with periampullary cancer, diagnostic laparoscopy may not be cost-effective. However, this has to be confirmed by other studies investigating the incidence of unresectability after direct laparotomy in patients with periampullary cancer or by further studies investigating the utility of diagnostic laparoscopy in patients with periampullary cancer.

### Strengths and weaknesses

The strengths of this study are that it was based on a recently published Cochrane review that analysed in detail the available evidence for the diagnostic accuracy of laparoscopy following CT scanning for assessing resectability in pancreatic and periampullary cancer.

An extensive sensitivity analysis has also been performed, which has been useful to show that the conclusions are sensitive to key model parameters surrounding test accuracy.

The main weakness of this study is that while the base case values show diagnostic laparoscopy is cost-effective, the sensitivity analysis indicates that the conclusions will change if key model parameters vary within feasible limits. Hence, conclusions surrounding the cost-effectiveness of diagnostic laparoscopy ought to be treated with caution and further research is recommended to assess cost-effectiveness in different settings. This ought to account for the sensitivity and specificity of diagnostic laparoscopy, the proportion of people unresectable after direct laparotomy, and the costs of laparotomy and diagnostic laparoscopy.

For simplicity the model has a time horizon of 6 months and only perioperative deaths are included. The underlying mortality rate in patients with pancreatic and periampullary cancer would affect both treatment arms equally, and since we are interested in differences in costs and outcomes between the two treatment arms changing the underlying mortality rate would have no impact on relative cost-effectiveness.

The costs associated with diagnostic laparoscopy prior to laparotomy versus direct laparotomy were the same; the QALY gains associated with diagnostic laparoscopy prior to laparotomy are statistically significantly different from zero, but small (mean QALY gain per patient 0.346 - 0.337 = 0.009). This difference may be less than the minimal clinically important difference (minimal clinically important differences in health state utility values are typically in the range 0.010 to 0.048) [26]. Hence, any gains from diagnostic laparoscopy prior to laparotomy purely in terms of QALYs may be misplaced.

### Comparison with other studies

This is the first study to evaluate the cost-effectiveness of diagnostic laparoscopy prior to laparotomy versus direct laparotomy in patients with pancreatic and periampullary cancer who were resectable following CT scanning.

### Implications for policy and practice

The implications of this study are that when laparotomy following diagnostic laparoscopy occurs in a subsequent admission, diagnostic laparoscopy appears to be cost-effective in decreasing unnecessary laparotomy in patients with pancreatic cancer (but not in those with periampullary cancer) found to have resectable disease on CT scan, producing a small improvement in health outcomes at no extra cost. Diagnostic laparoscopy with laparotomy undertaken in the same admission is not cost-effective.

The results are sensitive to the probability of unresectable disease (with curative intent) following CT scan that shows resectable disease and the accuracy of diagnostic laparoscopy. Given that both of these are operator dependent and the probability of unresectable disease (with curative intent following CT scan) may be lower in patients with periampullary cancer, it is recommended that these probabilities are studied over a period of time to ensure that the most cost-effective option is chosen in that particular setting.

Advances in imaging techniques such as refinements to CT, MRI or positron emission tomography (PET) scans alone or in combination may provide greater diagnostic sensitivity and specificity in pancreatic cancer. However, at present, MRI and PET scans are not as widely available or performed as CT scans and diagnostic laparoscopy and there is currently no evidence that MRI and PET scans decrease unresectability rates. The cost-effectiveness of diagnostic laparoscopy therefore should be revisited if there is evidence that refined CT scanning methods or routine MRI or PET scanning shows a reduction in unresectability rates.

### Further research

This study is based on a Cochrane review of the diagnostic accuracy of laparoscopy following CT scanning for assessing resectability in pancreatic and periampullary cancer. The review concluded that further diagnostic test accuracy studies with low risk of bias should be undertaken to calculate the utility of diagnostic laparoscopy more accurately. Given that the results in this study are sensitive to key model parameters for test accuracy, this research would also be beneficial for estimating whether or not diagnostic laparoscopy is cost-effective.

Further research would be useful to consider the impact that avoiding unnecessary laparotomies would have on freeing up operating theatre time and hospital beds if diagnostic laparoscopy was implemented into routine practice.

## Conclusions

Diagnostic laparoscopy in patients with CT resectable pancreatic and periampullary cancer appears to be cost-effective when laparotomy following diagnostic laparoscopy occurs in a subsequent admission.

## Additional file

Additional file 1:
**Model parameters for decision tree model and range of values used in univariate sensitivity analysis.**

## Abbreviations

CI: Confidence Intervals
CT: Computerised tomography
MNBs: Monetary net benefits
MRI: Magnetic resonance imaging
NHS: (UK) National Health Service
NICE: National Institute for Health and Care Excellence
PET: Positron Emission Tomography
PSA: Probabilistic sensitivity analysis
QALYs: Quality-Adjusted Life Years

## References

[1] Conlon KC, Klimstra DS, Brennan MF (1996). Long-term survival after curative resection for pancreatic ductal adenocarcinoma: clinicopathologic analysis of 5-year survivors. Ann Surg.

[2] Engelken FJ, Bettschart V, Rahman MQ, Parks RW, Garden OJ (2003). Prognostic factors in the palliation of pancreatic cancer. Eur J Surg Oncol.

[3] Michelassi F, Erroi F, Dawson PJ, Pietrabissa A, Noda S, Handcock M (1989). Experience with 647 consecutive tumors of the duodenum, ampulla, head of the pancreas, and distal common bile duct. Ann Surg.

[4] Shahrudin MD (1997). Carcinoma of the pancreas: resection outcome at the University Hospital Kuala Lumpur. Int Surg.

[5] Smith RA, Bosonnet L, Ghaneh P, Sutton R, Evans J, Healey P (2008). The platelet-lymphocyte ratio improves the predictive value of serum CA19-9 levels in determining patient selection for staging laparoscopy in suspected periampullary cancer. Surgery.

[6] Lillemoe KD, Cameron JL, Hardacre JM, Sohn TA, Sauter PK, Coleman J (1999). Is prophylactic gastrojejunostomy indicated for unresectable periampullary cancer? A prospective randomized trial. Ann Surg.

[7] Mayo SC, Austin DF, Sheppard BC, Mori M, Shipley DK, Billingsley KG (2009). Evolving preoperative evaluation of patients with pancreatic cancer: does laparoscopy have a role in the current era?. J Am Coll Surg.

[8] Allen VB, Gurusamy KS, Takwoingi Y, Kalia A, Davidson BR. Diagnostic accuracy of laparoscopy following CT scanning for assessing the resectability in pancreatic and periampullary cancer. Cochrane Database of Syst Rev. 2013, Issue 6. Art. No.: CD009323. doi:10.1002/14651858.CD009323.pub2. 10.1002/14651858.CD009323.pub2 24272022

[9] NHS Economic Evaluation Database. [http://www.crd.york.ac.uk/CRDWeb/]

[10] Morris S, Devlin N, Parkin D, Spencer A (2012). Economic analysis in health care.

[11] van Dijkum EJ N, Kuhlmann KF, Terwee CB, Obertop H, de Haes JC, Gouma DJ (2005). Quality of life after curative or palliative surgical treatment of pancreatic and periampullary carcinoma. Br J Surg.

[12] Schniewind B, Bestmann B, Henne-Bruns D, Faendrich F, Kremer B, Kuechler T (2006). Quality of life after pancreaticoduodenectomy for ductal adenocarcinoma of the pancreatic head. Br J Surg.

[13] Chan C, Franssen B, Dominguez I, Ramirez-Del Val A, Uscanga LF, Campuzano M (2012). Impact on Quality of Life After Pancreatoduodenectomy: A Prospective Study Comparing Preoperative and Postoperative Scores. J Gastrointest Surg.

[14] Wagner M, Redaelli C, Lietz M, Seiler CA, Friess H, Buchler MW (2004). Curative resection is the single most important factor determining outcome in patients with pancreatic adenocarcinoma. Br J Surg.

[15] Brooks AD, Mallis MJ, Brennan MF, Conlon KC (2002). The value of laparoscopy in the management of ampullary, duodenal, and distal bile duct tumors. J Gastrointest Surg.

[16] Krzyzanowska MK, Earle CC, Kuntz KM, Weeks JC (2007). Using economic analysis to evaluate the potential of multimodality therapy for elderly patients with locally advanced pancreatic cancer. Int J Radiat Oncol Biol Phys.

[17] Murphy JD, Chang DT, Abelson J, Daly ME, Yeung HN, Nelson LM (2012). Cost-effectiveness of modern radiotherapy techniques in locally advanced pancreatic cancer. Cancer.

[18] Glimelius B, Hoffman K, Graf W, Haglund U, Nyrén O, Påhlman L (1995). Cost-effectiveness of palliative chemotherapy in advanced gastrointestinal cancer. Ann Oncol.

[19] Karuna ST, Thirlby R, Biehl T, Veenstra D (2008). Cost-Effectiveness of Laparoscopy Versus Laparotomy for Initial Surgical Evaluation and Treatment of Potentially Resectable Hepatic Colorectal Metastases: A Decision Analysis. J Surg Oncol.

[20] Langenhoff BS, Krabbe PFM, Peerenboom L, Wobbes T, Ruers TJ (2006). Quality of life after surgical treatment of colorectal liver metastases. Br J Surg.

[21] National Schedule of Reference Costs - Year 2011–12 - NHS trusts and NHS foundation trusts: NHS own costs. [https://www.gov.uk/government/publications/nhs-reference-costs-financial-year-2011-to-2012]

[22] National Institute for Health and Care Excellence (NICE) Guide to the methods of technology appraisal 2013. [http://www.nice.org.uk/article/pmg9/chapter/Foreword] 27905712

[23] Briggs A, Sculpher M, Claxton K (2006). Decision modeling for health economic evaluation.

[24] Information Services Division Scotland. Theatre Services. [http://www.isdscotland.org/Health-Topics/Finance/Costs/Detailed-Tables/Theatres.asp]

[25] Pancreatic Section, British Society of Gastroenterology, Pancreatic Society of Great Britain and Ireland, Association of Upper Gastrointestinal Surgeons of Great Britain and Ireland, Royal College of Pathologists, Special Interest Group for Gastro-Intestinal Radiology (2005). Guidelines for the management of patients with pancreatic cancer periampullary and ampullary carcinomas. Gut.

[26] Walters S, Brazier J (2003). What is the relationship between the minimally important difference and health state utility values? The case of the SF-6D. Health Qual Life Outcomes.

